# Exposure to Abnormally Hot Temperature and the Demand for Commercial Health Insurance

**DOI:** 10.3389/fpubh.2022.842665

**Published:** 2022-01-25

**Authors:** Qian Zhong, Hao Zhang, Xiaoke Sun

**Affiliations:** ^1^Department of Financial Engineering, School of Finance, Guangdong University of Foreign Studies, Guangzhou, China; ^2^Department of Insurance, School of Finance, Guangdong University of Foreign Studies, Guangzhou, China

**Keywords:** abnormal high temperature, commercial health insurance, residents' health, global warming, Charls

## Abstract

Using the China Health and Retirement Longitudinal Study, this paper studies the impact of abnormal hot temperature on residents' demand for commercial health insurance. The results show that for every 1°F rise in abnormal temperature, the probability of people buying commercial health insurance increased by 6%. Furthermore, the abnormal hot temperature has a more significant impact on the commercial health insurance demand of women, residents in the South and residents in the East. Channel analysis shows that abnormal hot temperature affects the demand for commercial health insurance through two channels: increasing residents' concern about climate risk and affecting health. This paper provides evidence for actively promoting sustainable development and improving the construction of medical security system.

## Introduction

One hundred and seventy eight nations signed the Paris Agreement, committing to work together to fight global climate change in 2016. Studies show that climate change will not only harm the living environment of humans (1) but also affect investment behavior (2), social responsibility (3, 4), purchasing behavior (5, 6) and other economic activities. It is found that extreme temperatures have surged around the world in the past 20 years. For example, the Arctic, North Africa, Canada, and China's Qinghai-Tibet Plateau are warmer than before, while eastern and northern China are cooler. It is necessary to study the impact of extreme temperatures on human health and economic activities.

This paper explores the response of Chinese middle-aged and elderly people to abnormal temperatures. Specifically, we use household-level data come from China Health and Pension Tracking Survey (hereinafter Charls) in 2011, 2013 and 2015, to study whether the demand for commercial medical insurance of the elderly over 45 years old in Chinese provinces is related to abnormal temperature. We aim to explore whether abnormal temperature can be perceived by individuals and increase individuals' concerns about their own health.

Since human body temperatures remain constant, the abnormal temperature will affect human health by altering thermoregulatory responses (7), especially for the middle-aged and the elderly with poor physical performance (8). Abnormal high temperatures may cause sunstroke and neuropsychiatric disorders (9), and abnormal low temperatures may increase the risk of stroke (10). Extreme weather caused by abnormal temperatures also severely affects human life. For example, more than 30 tornadoes struck six states in the United States overnight on December 10, 2021, killing at least 90 people. Media reports about extreme weather events have brought climate change into focus and raised public concern on climate risks. Our paper focuses on the impact of abnormal temperature on the middle-aged and elderly, because the middle-aged and elderly are more sensitive to temperature. We use whether middle-aged and elderly people buy commercial medical insurance to measure their health concerns, and study whether abnormal temperature will increase the probability of middle-aged and elderly people buying commercial medical insurance.

We find that when the abnormal temperature increased by 1°F, the probability of middle-aged and elderly people buying commercial medical insurance increased by 6%, after controlling other factors that may affect the willingness of middle-aged and elderly people to buy commercial medical insurance. The influence of abnormally hot temperatures is more significant in eastern and southern cities. We also find that abnormal hot temperature does increase the prevalence rate of chronic diseases and lower self-rated health.

Our results are important for several reasons. First, this paper proves the necessity of promoting a green economy and sustainable development from the perspective of health economics. The United Nations proposed 17 sustainable development goals, including health and well-being, clean energy, sustainable consumption and production in 2015. In the past few years, many counties pursue economic development at the sacrifice of the environment. The carbon dioxide concentration in the atmosphere has increased by nearly 30 percent since the beginning of the Industrial Revolution (11), which is the main cause of global temperature anomalies. This article finds that abnormal hot temperatures harm human health and increase the probability of people buying commercial health insurance, which is echoed with earlier research that public concern about climate change raises when the local temperature is abnormally high. Therefore, clean energy is not only conducive to socially sustainable development, but also has positive externalities to other aspects, such as human health. The 17 sustainable development goals of the United Nations can complement and reinforce each other.

Second, we find new factors affecting individual commercial health insurance in China, which is very important due to the growing demand for commercial health insurance by Chinese residents. With the rapid development of China's national economy, the disposable income of residents is increasing. In recent years, Chinese people's health awareness has been strengthened. People pay more and more attention to the maintenance of daily health and the prevention and treatment of diseases. Medical spending accounted for a large percentage of total household consumption in China, with a proportion of 8.63% in 2020^1^. It is necessary to study the key elements influencing the demand for individual commercial health insurance. The existing literature focuses on the impact of air quality on individual health insurance demand (12), because a large number of literature find that air pollution affects people's health (13) and even increases people's mortality (14). This paper confirms that abnormal high temperature also affects people's demand for commercial health insurance because abnormal high temperatures will not only raise people's concerns about climate risks, but also affect people's health.

Third, our work provides evidence that climate risk affects individual health insurance decisions. Climate risk directly affects crops, thus a large number of insurance related literature focus on whether crop insurance (15) or weather index insurance (16) can minimize farmers' financial losses in the event of extreme climate risk. However, there is no literature on the relationship between climate risk and residents' commercial health insurance in the field of economics, even though many medical studies have found that abnormal temperature does affect residents' physical (17–22) and mental health (23). Our results show that people can feel the abnormal temperature and worry about the impact of abnormal temperature on their own health. Therefore, abnormal high temperature will increase the probability of people buying commercial health insurance. Our work advances existing research on how people respond to climate risks.

## Literature Review and Hypotheses

Extreme weather events cause severe economic losses and even lead to human death. More and more people pay attention to extreme climates and their causes (24). Many kinds of literature believe that Carbon emissions are one of the main drivers of global warming (25). China has adopted economic policies such as green credit and green subsidies, to encourage energy conservation and renewable resources development (26, 27). If the public can recognize the harm of the greenhouse or worry about climate risks, they will consciously abide by the carbon reduction policy. Conversely, when people feel difficulty in recognizing the greenhouse effect, they will not follow that policy as higher compliance costs.

Some population surveys find that many adults can indeed feel global warming after experiencing climate change (28). People feel changes in seasons, weather, lake levels, snowfall, plants and animals (29). Building psychological models, some studies find that residents are highly concerned with abnormal temperature and concern about global warming (30). Based on American survey data, Konisky et al. (31) show that extreme weather experiences will raise fears of climate risks. It is well-known that insurance is an economic compensation method for dispersing risks and compensating losses. As extreme weather activities cause individuals' concern about climate risk, will it increase their willingness to buy insurance?

In fact, many kinds of research in agriculture indicate that agricultural insurance is a powerful instrument to manage climate risks (32). Climate change affects the average atmospheric conditions, the variability of weather conditions, and the frequency of extreme weather events, further affecting crop productivity and the welfare of small farmers. So agricultural insurance against extreme temperatures is very necessary (33). However, there is little research on the impact of climate risks on other insurance demands, especially health insurance.

A large number of medical studies find that extreme temperatures can lead to an increase in morbidity and mortality (17–19). The heatwave that occurred in Europe in 2003 caused up to 30,000 deaths (20). During the 2010 heatwave in Nanjing, China, the stroke mortality rate was also higher than the same period in previous and subsequent years (21). Van Loenhout et al. (22) find exposure to high ambient temperatures leads to an increase in mortality and morbidity, especially in the elderly, as the health status of the elderly is poor (8) and they can barely walk (34). Medical studies confirm that excessive temperature can cause thirst, sleep disturbance and excessive perspiration in the elderly (22). The extreme temperature will also increase the probability of stroke or cardiovascular disease (35). Some studies find that urogenital diseases may be related to extreme temperatures (36, 37).

Based on the above analysis, it can be seen that the middle-aged and the elderly are aware of global warming, and they pay more attention to climate risks than young people (38). We can also see that the health status of the middle-aged and the elderly is more vulnerable to extreme temperatures (39). As the living standard rises, Life expectancy is increasing. Compared with young people, the middle-aged and the elderly are more concerned about their old-age arrangements and health insurance. When the middle-aged and the elderly obviously feel that their health is affected by extreme temperatures, they may purchase commercial health insurance to avoid potential climate risks. Therefore, we propose:

Hypothesis: *Abnormal temperature will increase the probability of the middle-aged and the elderly buying commercial health insurance*.

## Research Design

### Data

The data of this paper consists of two parts. In the following, we introduce the databases we use, as well as the variables we obtain and examine in our analyses.

### Temperature Data

We obtain daily temperature data of China from the Global Surface Summary of Day Data, produced by the National Climatic Data Center (NCDC). For our analysis, we collected daily temperature data from various provinces in China. Specifically, we selected the weather data of the weather station nearest to the provincial capital to represent the weather data of the province.

### Insurance Demand and Family Information

We obtained various personal information of middle-aged and elderly people in Charls. Charls aims to collect a set of high-quality micro data representing families and individuals of middle-aged and elderly people aged 45 and over in China for interdisciplinary research on aging. Charls national baseline survey was carried out in 2011, covering 17000 people in about 10,000 households in 150 county-level units. Charls conducted follow-up surveys on these families in 2013, 2015 and 2018. We used Charls' data for 2011, 2013 and 2015. The advantage of using this data is that Charls is a survey data specifically for people over 45 years old, covering individual health, income, education, and whether to buy social insurance and commercial insurance, which meets the requirements of this study.

### Research Design

Refer to the previous literature on the demand for individual commercial health insurance (40), we establish the following logit model:


(1) $$\begin{array}{r} logit\left(Insurance_{it}\right)=\beta_{1}abt_{it}+\beta_{2}Controls_{it}+\varepsilon_{it} \end{array}$$

*Insurance*_*it*_ is a dummy variable that respondents in the Charls questionnaire answer whether they purchase commercial health insurance this year. When individuals purchase commercial health insurance, it is 1, otherwise it is 0.

*abt*_*it*_ is the abnormal temperature. Referring to Choi et al. (2), Garel and Petit-Rromec (4), we divide the local temperature into three dimensions: the predictable part according to the historical temperature, the seasonally adjusted part and the abnormal part:


(2) $$\begin{array}{r} {temperature}_{jt}={aver}_{jt}+mont_{jt}+abt_{jt} \end{array}$$

*temperature*_*it*_ is the actual monthly average temperature in month t of province j. *aver*_*jt*_ is the average temperature in the past 120 months of province j, which measures the temperature that can be predicted according to the historical temperature. *mont*_*jt*_ is the average value of the difference between the temperature in the past 120 months of province j and *aver*_*jt*_, in other words, the average value of the temperature deviation, to measure the seasonal adjustment. *abt*_*jt*_ equals to the actual temperature minus *aver*_*jt*_ and *mont*_*jt*_.Since the micro individual data is annual data, following Garel and Petit-Romec (4), we average the monthly abnormal temperature data in the past 12 months as the abnormal temperature data experienced by the middle-aged and elderly in the past year.

*Controls*_*i,t*_ is a series of control variables at the individual level, including demographic variables such as age, gender and marital status, socio–economic status variables such as education and family per capita income, and health variables such as whether there is hypertension and the number of chronic diseases. ε_*it*_ represents the disturbance term. The description of each variable is shown in Table 1.

**Table 1:** Description of variables.

**Variable**	**Description**
pcmi	Whether the respondent has personal commercial medical insurance
abt	Following Choi et al. (2) and Garela and Petit-Romec (4), we first calculate the monthly abnormal temperature according to formula (1), then average the monthly abnormal temperature over the last twelve months.
Age	Age of respondent.
Gender	A dummy variable representing the gender of the respondent. Take 1 when the respondent is male and 2 when the respondent is female
married	The dummy variable indicating the marital status of the respondent. If married and the spouse is still alive, the value is 1, otherwise it is 0.
Edu_Group	A dummy variable indicating the education level of the respondent. It is 1 for illustrate, 2 for literal, 3 for primary, 4 for middle, and 5 for high and above.
lnfainc	Logarithm of per capita household income of respondent.
chro_disease_num	Number of chronic diseases of the respondent.
hypertension	The dummy variable to measure whether the respondent has hypertension, 1 when the respondents have hypertension, otherwise 0.

## Empirical Results and Analysis

### Descriptive Statistics

Table 2 reports the descriptive statistics of the main variables in this paper. During the sample period, only 1.96% of the middle-aged and elderly people purchased commercial medical insurance, which shows that the coverage rate of commercial medical insurance in China is very low. Medical insurance in China is a complementary system of social medical insurance and commercial medical insurance. Social medical insurance mainly undertakes the responsibility of basic medical resources under the fair distribution (41), but the coverage rate of social medical insurance is far lower than that of Germany. As a supplement to social medical insurance, commercial medical insurance is more responsible for some optional and personalized medical services. The low coverage rate of commercial medical insurance in China during the sample period shows not only the lack of public security awareness, but also the lack of economic development in China.

**Table 2:** Descriptive statistical analysis.

**Variable**	**Mean**	**Standard deviation**	**Min**	**Max**
pcmi	0.0196	0.1388	0	1
abt	−0.2128	1.0305	−5.1735	14.8848
Age	59.5686	9.7918	45	101
Gender	1.5139	0.4998	1	2
married	0.8696	0.3368	0	1
Edu_Group	2.8660	1.3581	1	5
lnfainc	8.1318	2.6127	0	17.3720
chro_disease_num	1.5207	1.5037	0	11
hypertension	0.3936	0.4885	0	1

The mean value of *abt* is −0.21°F, but the standard deviation is 1.03°F. The maximum abnormal temperature is 14.88°F and the minimum abnormal temperature is −5.17°F, indicating that there are great differences in abnormal temperatures among provinces, due to the wide area of China. During the sample period, the abnormal temperature in most provinces is negative, which may be related to the cold wave in China in 2012.

The mean value of *Age* is 59.56, indicating that the average age of Charls respondents is close to 60. The mean value of *Gender* is 1.51, showing that the male to female ratio is close to 1:1. Most of the interviewees are married as the average value of *married* is 0.87. The mean value of *Edu_ group* is 1.35, means that the education level of most elderly people in China is very limited, even a considerable proportion of elderly people are illiterate. There is a large gap between the rich and the poor among the middle-aged and elderly in China as the standard deviation is 2.61. The mean value of *chro_disease_number* is 1.52, shows that the middle-aged and elderly people suffer from 1.5 kinds of chronic diseases. old suffer from 1.5 kinds of chronic diseases on average 0.39% of the middle-aged and elderly people have hypertension because the mean value of hypertension is 0.39.

### Baseline Regressions

Table 3 shows the regression results of Formula 1. Column 1 of Table 3 shows the regression results under the control of demographic variables. When the abnormal hot temperature increases by 1°F, the probability of buying commercial health insurance increases by 5.6%, which is significant at the significance level of 10%. Column 2 is the regression result after controlling demographic variables and socio-economic status variables. The influence coefficient of abnormal temperature on individual's demand for commercial health insurance is 0.0665. Column 3 is the regression result after controlling demography, socioeconomics and health level. The results showed that the demand for commercial health insurance increased by 6.76% for every 1°F increase in abnormal high temperature, and the result was significant at the 5% significance level.

**Table 3:** The impact of abnormal temperature on the demand of commercial medical insurance.

	**(1)**	**(2)**	**(3)**
abt	0.0566^*^	0.0665^*^	0.0678^**^
	(0.034)	(0.034)	(0.034)
Age	−0.0935^***^	−0.0795^***^	−0.0824^***^
	(0.005)	(0.006)	(0.006)
Gender	−0.0581	0.1776^**^	0.1670^**^
	(0.074)	(0.078)	(0.078)
married	−0.1568	−0.2550^*^	−0.2494^*^
	(0.138)	(0.138)	(0.139)
Edu_Group_Literate		0.3491^*^	0.3389^*^
		(0.186)	(0.186)
Edu_Group_ Primary		0.6668^***^	0.6608^***^
		(0.158)	(0.158)
Edu_Group_Middle		1.0082^***^	1.0039^***^
		(0.154)	(0.154)
Edu_Group_High and Above		1.6420^***^	1.6391^***^
		(0.154)	(0.154)
lnfainc		0.0269	0.0280^*^
		(0.017)	(0.017)
chro_disease_num			0.0649^**^
			(0.025)
Constant	1.5575^***^	−0.5601	−0.4860
	(0.364)	(0.456)	(0.454)
R2	0.0797	0.0806	0.0805
Observations	38,711	38,711	38,711

***, ** and ^*^ represent significance levels of 1%, 5% and 10%, respectively. The numbers in brackets are standard errors.

In addition, it can be seen from Table 3 that the coefficient of *Age* is significantly negative, which indicates that the older the residents are, the lower the probability of purchasing commercial health insurance, which is related to the higher premium of commercial health insurance for the older people. The coefficient of *Gender* is significantly positive, showing that abnormal hot temperature has a greater impact on women's health insurance demand. Compared with divorced or widowed middle-aged and elderly people, the middle-aged and elderly people with normal marriage have less demand for commercial health insurance, as the coefficient of *married* is significantly negative, which may be because the middle-aged and elderly people with normal marriage have stronger happiness. The more educated people are, the more likely they are to buy commercial health insurance due to abnormal high temperature. It shows that education improves individual health risk awareness and climate risk awareness, which is consistent with the results of existing literature (42). Consistent with practical experience, people with higher income have more demand for commercial health insurance, and people with worse physical condition have higher probability of purchasing commercial health insurance.

### Robustness Analysis

In order to confirm the reliability of the above conclusions, the following robustness tests are carried out in this paper. The results are shown in Table 4. The original research conclusions are robust.

**Table 4:** Robustness test.

	**(1)**	**(2)**	**(3)**	**(4)**	**(5)**
	**Probit**	**More control variables**	**IV**	**another abnormal temperature**	**Lagging abnormal temperature**
abt	0.0297^**^	0.0605^*^	0.3228^***^	0.0322^*^	0.0798^**^
	(0.015)	(0.035)	(0.099)	(0.017)	(0.034)
Age	−0.0329^***^	−0.0844^***^	−0.0343^***^	−0.082^***^	−0.0826^***^
	(0.002)	(0.006)	(0.002)	(0.006)	(0.006)
Gender	0.0725^**^	0.1660^**^	0.0706^**^	0.1679^**^	0.1659^**^
	(0.033)	(0.081)	(0.033)	(0.078)	(0.078)
married	−0.1077^*^	−0.3075^**^	−0.1178^**^	−0.2456^*^	−0.2533^*^
	(0.057)	(0.141)	(0.057)	(0.138)	(0.139)
Edu_Group_Literate	0.1232^*^	0.3003	0.1454^**^	0.3342^*^	0.3423^*^
	(0.070)	(0.194)	(0.071)	(0.185)	(0.186)
Edu_Group_ Primary	0.2551^***^	0.6083^***^	0.2356^***^	0.6716^***^	0.6559^***^
	(0.059)	(0.164)	(0.060)	(0.159)	(0.158)
Edu_Group_Middle	0.3896^***^	0.9342^***^	0.3755^***^	1.010^***^	0.9981^***^
	(0.058)	(0.161)	(0.060)	(0.153)	(0.154)
Edu_Group_High and Above	0.6709^***^	1.5355^***^	0.6793^***^	1.633^***^	1.6365^***^
	(0.060)	(0.163)	(0.061)	(0.154)	(0.154)
lnfainc	0.0118^*^	0.0221	0.0154^**^	0.0269	0.0287^*^
	(0.007)	(0.017)	(0.006)	(0.017)	(0.017)
chro_disease_num	0.0263^**^	0.0903^***^	0.0255^**^	0.0653^***^	0.0647^***^
	(0.011)	(0.028)	(0.011)	(0.0250)	(0.025)
body_pain		−0.1923^**^			
		(0.095)			
memory_poor		−0.2540^**^			
		(0.099)			
times_hospital		0.0334			
		(0.070)			
Constant	−0.6778^***^	−0.1466	−0.5474^***^	−0.5120	−0.4687
	(0.183)	(0.470)	(0.180)	(0.454)	(0.455)
R2	0.0802	0.0823		0.0803	0.0808
Wald test Prob			0.0027		
Observations	38,711	36,450	38,711	38711	38688

***, ** and ^*^ represent significance levels of 1%, 5% and 10%, respectively. The numbers in brackets are standard errors.

First, replace logit model with probit model to re estimate. The results are shown in Table 4 Column 1. When we use probit model to estimate the impact of abnormal temperature on individual health insurance demand, we can still get the conclusion that abnormal hot temperature will increase the probability of individual purchasing commercial health insurance, which is significant at the 5% confidence level. However, the coefficient of abnormal temperature on purchase probability estimated based on probit model is slightly smaller than that estimated based on logit model.

Second, include more health-related control variables. Some medical studies find that the effect of abnormal temperature on mortality is often affected by personal medical history and health status (43). In order to reduce the impact of personal health status on individuals' demand for commercial health insurance, we try to control more personal health status variables, including physical pain, good memory and the number of hospitals visits last year. After more control variables are included, we use logit model for regression, and the results are shown in Column 2 of Table 4. The influence coefficient of abnormal temperature on the purchase probability of individual commercial health insurance is 0.0605, which is close to the benchmark regression result.

Third, use tool variables. The behavior of individuals' buying commercial health insurance can not affect the occurrence of abnormal temperature. So, there is no reverse causality in the regression of this paper. However, we are still worried about the possibility that the positive relationship between abnormal temperature and the probability of purchasing commercial health insurance is due to a common trend rather than causality. In order to eliminate this potential possibility, we use the carbon emissions of each province as a tool variable to do 2SLS regression. The reason for using carbon emission as a tool variable is that the higher the carbon emission, the greater the heat island effect and the greater the probability of abnormal temperature. However, carbon dioxide itself is not toxic, so it will not directly affect human health or the probability of human purchasing commercial health insurance. Therefore, we believe that carbon emission is an effective instrumental variable. The regression results of using instrumental variables are shown in column 3 of Table 4. The influence coefficient of abnormal temperature on the probability of individual purchasing commercial health insurance is still significantly positive.

Fourth, change the measurement method of abnormal temperature^2^. Here, we use the difference between the annual average temperature in the sample period and the historical average temperature as the abnormal temperature. We use the average temperature of each province from 1990 to 1999 to measure the historical average temperature. After changing the measurement method of abnormal temperature, the regression results are shown in column 4 of Table 4. Our conclusion is still robust: abnormal high temperature increases the probability of people buying commercial health insurance.

Fifth, use the abnormal temperature with a lag period. People's perception of global warming and abnormal temperature is a process, and people's purchase of commercial insurance is also an annual decision. Therefore, there may be a time lag in the impact of abnormal temperature on people's decision to buy commercial health insurance. We use the abnormal temperature of lag phase I for logit regression. The regression results are shown in column 5 of Table 4. Our conclusion is still valid.

### Supplementary Analysis

Abnormal temperature will affect the probability of human morbidity and cause human concern about their own health, so it increases the probability of individuals buying commercial health insurance. Is this logic correct? For further verification, we use a two-way fixed effect model that controls both individual and time effects to estimate the impact of abnormal temperature on individual health. Medical research (44) uses potential cases calling emergency calls to study the impact of abnormal temperature on health. However, there was sample selection error. Using Charls, a nationwide sampling questionnaire data, can reduce sample selection error. As shown in Table 5, we used individual self-evaluation of health status, hypertension and the number of chronic diseases to measure individual health status, The regression results are listed in columns 1–3 respectively. It can be seen from Table 5 that the higher the abnormal hot temperature, the lower people's self-evaluation of their own health, which shows that the middle-aged and elderly people can really feel the abnormal temperature and worry about climate risk (31), and they also clearly feel the negative impact of abnormal temperature on their health. Although abnormal temperature does not increase the probability of hypertension in the middle-aged and elderly, it significantly increases the number of chronic diseases in the middle-aged and elderly, which is consistent with the conclusions of most medical studies (8, 36, 37).

**Table 5:** The impact of abnormal temperature on health.

	**(1)**	**(2)**	**(3)**
	**Self-rated health**	**Hypertension**	**Number of chronic diseases**
abt	−0.0284^***^	0.0037	0.0641^***^
	(0.003)	(0.003)	(0.008)
Controls	Yes	Yes	Yes
Individual & Year	Yes	Yes	Yes
R-squared	0.162	0.169	0.257
Observations	38,911	38,911	38,911

*** *represent significance levels of 1%, 5% and 10%, respectively. The numbers in brackets are standard errors*.

## Further Study

### Heterogeneity Analysis

All over the world, women live longer than men, both in developed and developing countries, with a mean difference of 4.2 years (45). There are great differences in men's and women's living habits, risk awareness and thinking mode. Therefore, the samples are divided into men and women for research respectively. The results are shown in columns 1 and 2 of Table 6. Abnormal hot temperature significantly increased the probability of middle-aged and elderly women buying commercial health insurance, but had no significant effect on the probability of men buying commercial health insurance. This shows that women are more willing to use insurance to reduce the possible health deterioration and financial vulnerability caused by abnormal hot temperature, in other words, women appear to be more financially risk averse than men (46).

**Table 6:** Heterogeneity analysis.

	**(1)**	**(2)**	**(3)**	**(4)**	**(5)**	**(6)**
	**Male**	**Female**	**Eastern city**	**Non Eastern**	**Southern cities**	**Non Southern**
abt	0.0581	0.0806^*^	0.1002^*^	0.0126	0.1155^**^	0.0245
	(0.049)	(0.047)	(0.055)	(0.046)	(0.053)	(0.048)
Control	Yes	Yes	Yes	Yes	Yes	Yes
R2	0.0827	0.0833	0.0787	0.0830	0.0769	0.0778
Observations	18,848	19,863	16,399	22,312	19,874	18,837

** and ^*^ represent significance levels of 1%, 5% and 10%, respectively. The numbers in brackets are standard errors.

Urban Heat Island (UHI) intensity is affected by the characteristics of the urban environment, longitude and latitude (47), so the influence of abnormal temperature may vary from province to province. We divide the samples into eastern and non-eastern cities, southern and northern cities, and the regression results are listed in columns 3 and 4, and 5 and 6 of Table 6, respectively. It can be seen from the results that the abnormal hot temperature in eastern cities and southern cities significantly increases the probability of individuals purchasing commercial health insurance.

### Channel Analysis

From the literature review, we know that middle-aged and elderly people can perceive climate change and global warming. On the other hand, abnormal hot temperature will affect individual health. Therefore, middle-aged and elderly people will purchase commercial health insurance to avoid potential financial vulnerabilities caused by abnormally hot temperatures. The following sections will discuss the existence of two channels, climate risk concern and health.

Abnormally hot temperatures increase the demand for commercial health insurance by increasing people's concerns about climate risks. Humans are more likely to purchase commercial health insurance when they clearly feel the dangers of global warming and abnormally hot temperatures to human health. Therefore, we divide the sample into two groups for regression according to whether the interviewee's cognition is abnormal. The results are shown in column 1 and 2 of Table 7. Abnormally hot temperature significantly increases the probability of purchasing health insurance for individuals without cognitive impairment, while there is no significant relationship between abnormally hot temperature and purchasing commercial health insurance in individuals with cognitive impairment. This verifies the existence of channel for climate risk concerns. Studies find that temperature affects the prevalence of depression and sadness: the prevalence of depression is highest in summer and autumn (48). Psychiatric patients may be more vulnerable to high temperatures (23), which under current climate change projections will most likely increase the concerns of people in poor mental states. Bundo et al. (23) find that increasing temperatures could negatively affect mental status in psychiatric patients using a 45-year time-series study. Thus, compared with normal people, patients with depression are more likely to do something to protect themselves from climate risks. Buying commercial health insurance is a very effective means. We divide the sample into two groups for regression according to whether the respondents have depressive tendency. The results are shown in the columns 3 and 4 of Table 7. Abnormal hot temperature significantly increases the probability of individuals with depression tendency to buy commercial health insurance, which corresponds to Huibers et al. (48). The regression results once again prove that concern about climate risk is one of the channels through which abnormally hot temperatures affect individual commercial health insurance needs.

**Table 7:** Mental state and perception.

	**(1)**	**(2)**	**(3)**	**(4)**
	**cognitive normal**	**Cognitive impairment**	**Non-severe depression**	**Severe depression**
abt	0.1510^***^	−0.0940	0.0166	0.1219^***^
	(0.041)	(0.058)	(0.048)	(0.047)
Controls	Yes	Yes	Yes	Yes
R2	0.0893	0.0727	0.0805	0.0826
Observations	23,145	15,566	21,912	16,799

*** *represent significance levels of 1%, 5% and 10%, respectively. The numbers in brackets are standard errors*.

Abnormal temperature also affects human health and increases individual demand for commercial health insurance. To test this channel, we divided the samples into healthy and unhealthy groups for regression according to the individual's evaluation of self-health, The regression results are shown in Column 1 and 2 of Table 8. People with unhealthy self-assessment pay more attention to the factors that may affect their own health, so the abnormal hot temperature significantly increases the probability of purchasing commercial health insurance for people with unhealthy self-assessment. On the contrary, people with healthy self-assessment are more confident in their own physical quality, they are not afraid of the potential impact of abnormal high temperature on their health. We also divide the sample into Obese and non-obese groups according to the BMI index. Obese people have poor health and are more afraid of heat (49), so we expect that abnormal hot temperature will have a more significant impact on obese people. The regression results are listed in columns 3 and 4 of Table 8, which are in line with expectations and further prove the establishment of health channel.

**Table 8:** Physical health.

	**(1)**	**(2)**	**(3)**	**(4)**
	**Healthy**	**Unhealthy**	**Fat**	**Nonobese**
abt	0.0504	0.0725^*^	0.0676^*^	0.0682
	(0.072)	(0.039)	(0.039)	(0.066)
Controls	Yes	Yes	Yes	Yes
R2	0.0738	0.0813	0.0777	0.0847
Observations	6,434	32,277	23,605	15,106

* *represent significance levels of 1%, 5% and 10%, respectively. The numbers in brackets are standard errors*.

## Conclusion

Based on Charls, this paper studies the impact of abnormal hot temperature on the demand for commercial health insurance for the middle-aged and elderly in China. We find that the higher the abnormal hot temperature, the lower the self-evaluation of personal health, the higher the number of chronic diseases, and the greater the probability of purchasing commercial health insurance. Heterogeneity analysis shows that abnormal hot temperature has a more significant impact on the commercial health insurance needs of women, southern urban residents and eastern urban residents. Channel analysis shows that abnormal temperature affects the probability of residents purchasing commercial health insurance through two channels: increasing residents' attention to climate risk and directly affecting residents' health.

Our research shows that the impact of abnormal hot temperature on human health has been noticed. People try to use financial instruments to avoid the impact of health risks under abnormal hot temperature on finance. Therefore, sustainable development is not only conducive to the survival of future generations, but also conducive to the health of contemporary mankind.

Secondly, health insurance, especially social health insurance, should consider bringing the health risks brought by global warming into the scope of protection. It is found that abnormal hot temperature significantly affects human health. Relevant departments should pay attention to the coverage of diseases caused by abnormal hot temperature in order to improve social welfare and promote social equity.

## Data Availability Statement

The original contributions presented in the study are included in the article/Supplementary Material, further inquiries can be directed to the corresponding author.

## Author Contributions

QZ contributed to the idea, completed the empirical test and analysis, and wrote the manuscript. HZ gave some important suggestions. XS performed the data analyses and manuscript preparation. All authors contributed to the article and approved the submitted version.

## Funding

This study is supported by the Guangdong University of Foreign Study (Project Nos. 21QN22 and 21QN08).

## Conflict of Interest

The authors declare that the research was conducted in the absence of any commercial or financial relationships that could be construed as a potential conflict of interest.

## Publisher's Note

All claims expressed in this article are solely those of the authors and do not necessarily represent those of their affiliated organizations, or those of the publisher, the editors and the reviewers. Any product that may be evaluated in this article, or claim that may be made by its manufacturer, is not guaranteed or endorsed by the publisher.

## Footnotes

^1^Data resource: CEIC datebase.

^2^Thank the reviewers for their valuable comments.
